# The Lipolysome—A Highly Complex and Dynamic Protein Network Orchestrating Cytoplasmic Triacylglycerol Degradation

**DOI:** 10.3390/metabo10040147

**Published:** 2020-04-10

**Authors:** Peter Hofer, Ulrike Taschler, Renate Schreiber, Petra Kotzbeck, Gabriele Schoiswohl

**Affiliations:** 1 Institute of Molecular Biosciences, University of Graz, 8010 Graz, Austria; peter.hofer@uni-graz.at (P.H.); ulrike.taschler@uni-graz.at (U.T.); renate.schreiber@uni-graz.at (R.S.); 2 Division of Endocrinology and Diabetology, Department of Internal Medicine, Medical University of Graz, 8036 Graz, Austria; petra.kotzbeck@medunigraz.at

**Keywords:** lipolysis, adipose tissue, triacylglycerol, lipase, fatty acid, lipid biochemistry, lipid droplets

## Abstract

The catabolism of intracellular triacylglycerols (TAGs) involves the activity of cytoplasmic and lysosomal enzymes. Cytoplasmic TAG hydrolysis, commonly termed lipolysis, is catalyzed by the sequential action of three major hydrolases, namely adipose triglyceride lipase, hormone-sensitive lipase, and monoacylglycerol lipase. All three enzymes interact with numerous protein binding partners that modulate their activity, cellular localization, or stability. Deficiencies of these auxiliary proteins can lead to derangements in neutral lipid metabolism and energy homeostasis. In this review, we summarize the composition and the dynamics of the complex lipolytic machinery we like to call “lipolysome”.

## 1. Introduction

**Figure 1:**
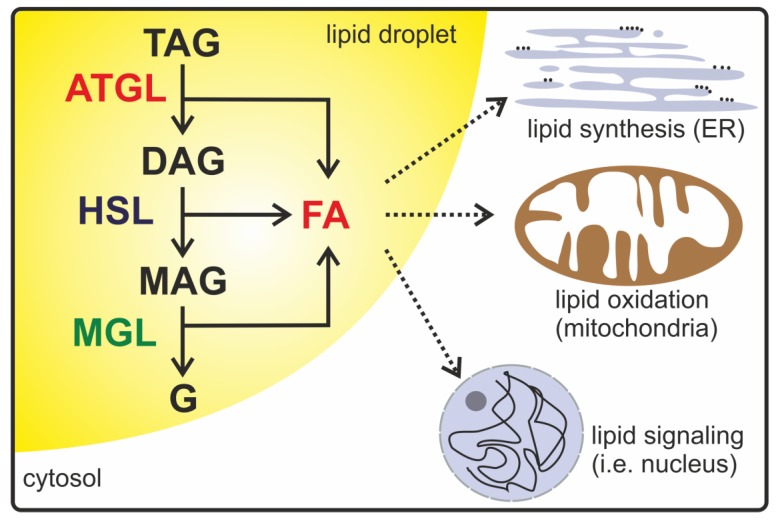
A schematic description of intracellular lipolysis. In the lipolytic active state, adipose triglyceride lipase (ATGL), hormone-sensitive lipase (HSL), and monoacylglycerol (MAG) lipase (MGL) are localized on the surface of lipid droplets in the cytoplasm. ATGL catalyzes the initial step in triacylglycerol (TAG) hydrolysis, generating diacylglycerol (DAG) and fatty acids (FAs). HSL preferentially degrades DAG into MAG and FAs. In the last step, MGL releases FAs and glycerol (G). Lipolytic products such as DAG, MAG, FAs, and glycerol are used as energy resources, signaling lipids, or precursors for lipid synthesis and membrane biosynthesis.

The stepwise enzymatic hydrolysis of intracellular triacylglycerols (TAGs) is a central biochemical process occurring in virtually every cell or tissue type. Depending on the origin of TAGs, two distinct pathways mediate their degradation [1]. Lipoprotein-derived TAGs are mostly delivered to lysosomes and hydrolyzed by lysosomal acid lipase at acidic pH, while TAGs that are stored in cytoplasmic lipid droplets (LDs) are predominantly hydrolyzed by cytosolic enzymes at neutral pH. Depending on the cell type, autophagy and lysosomal hydrolysis can also contribute to the degradation of LD-associated TAGs [2]. Lipolytic products, including diacylglycerols (DAGs), monoacylglycerols (MAGs), fatty acids (FAs), and glycerol, serve as energy resources, building blocks for lipid remodeling, or membrane biosynthesis, and signaling molecules influencing gene transcription or enzyme activity (Figure 1) [3,4]. Considering these pleotropic functions, it is obvious that lipolysis needs to be tightly controlled to ensure metabolic homeostasis. As a consequence, dysregulated or dysfunctional lipolysis is associated with severe pathological conditions, including obesity, lipodystrophy, cardiovascular disease, insulin resistance, fatty liver disease, cancer, or cancer-associated cachexia [5].

**Figure 2:**
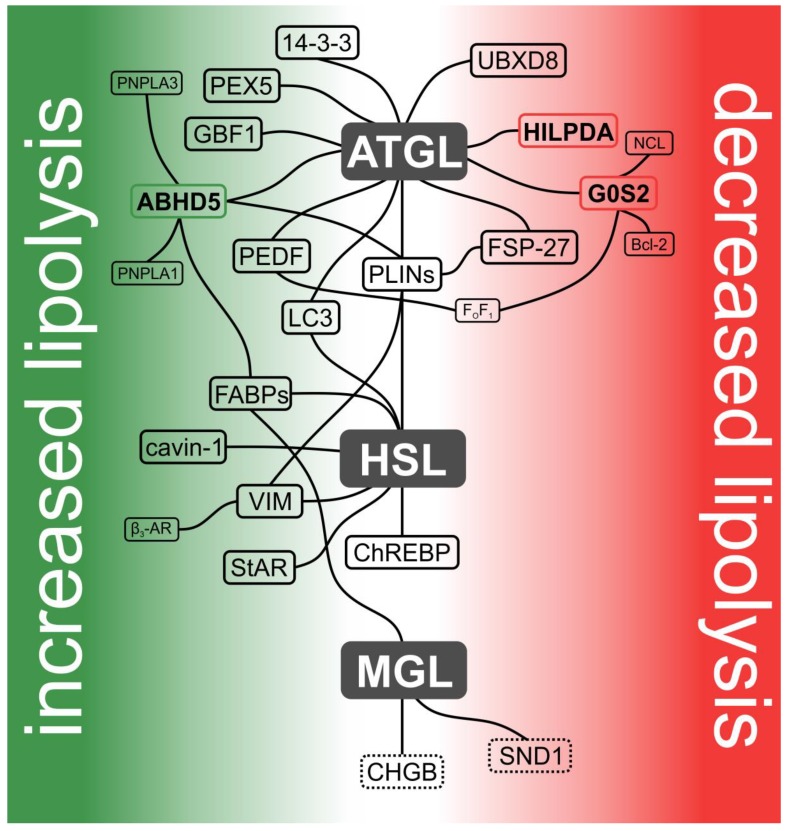
Current model of the lipolysome. Hydrolysis of intracellular TAGs is catalyzed by the sequential action of ATGL, HSL, and MGL. Together with their protein interaction partners, these lipases form a dynamic protein assembly designated as the lipolysome. Interaction partners that activate enzyme activities are depicted in the green area, while those that inhibit enzyme activities are shown in the red area. Proteins directly affecting enzyme activity are highlighted in bold and in colored boxes accordingly (in green: activating and in red: inhibiting). Proteins indirectly affecting lipase activities are shown in black boxes. Putative binding partners are shown in dashed boxes. Lipase-independent interaction partners are shown in smaller font. Interaction partners not affecting lipase activity per se (CHGB (chromogranin B) and carbohydrate response element binding protein (ChREBP)) or having divergent effects on ATGL and HSL (perilipins (PLINs)) are located in the center.

The three major enzymes facilitating cytoplasmic lipolysis are adipose triglyceride lipase (ATGL), hormone-sensitive lipase (HSL), and MAG lipase (MGL). Studies on the spatial and temporal regulation of these enzymes revealed unexpected complexity by uncovering ATGL, HSL, and MGL protein interaction partners modulating enzyme activity, localization, stability, or affinity to other proteins. Deficiency of many of these auxiliary proteins leads to pronounced alterations in neutral lipid metabolism and associated processes [6,7,8,9,10]. Thus, intracellular lipolysis, equivalently to gastrointestinal lipolysis [11] or lipogenesis [12], depends on the interactions of its enzymes with a broad set of regulatory proteins. The term “lipolysome”—initially introduced by Rudolf Zechner—describes the complexity of the lipolytic machinery. Here, we concisely summarize the current knowledge about the molecular composition and functionality of the lipolysome (Figure 2).

## 2. ATGL and Its Interaction Partners

**Figure 3:**
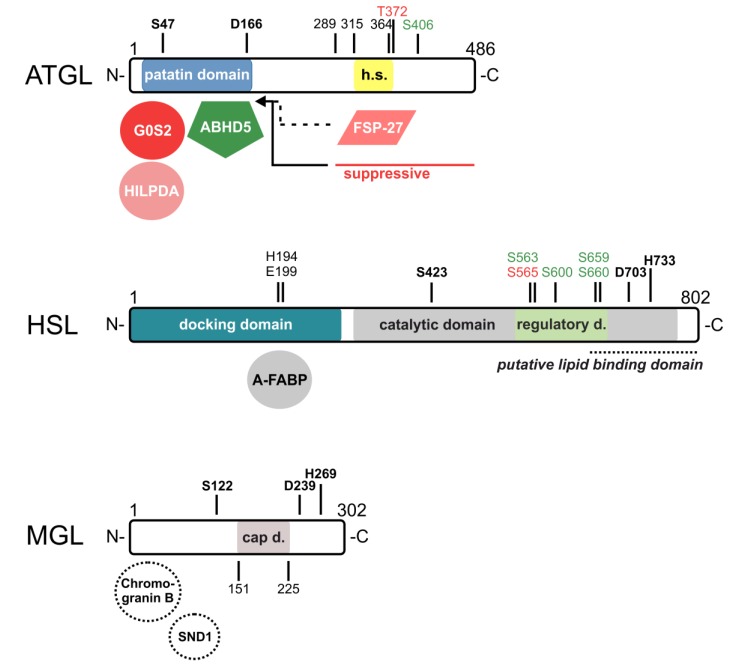
Domain structures and identified sites of (putative) interaction partners of ATGL, HSL, and MGL. For murine ATGL, the catalytic dyad consists of S47 and D166 within the patatin domain at the N-terminus. This domain serves as a docking domain for the co-activator alpha/beta-hydrolase domain containing protein 5 (ABHD5) and the inhibitors G0/G1 switch gene 2 (G0S2) and hypoxia-inducible LD-associated protein (HILPDA) of ATGL. At the C-terminus, ATGL contains a hydrophobic stretch (h.s.) from amino acids 315–364 that is essential for lipid droplet (LD) localization. The phosphorylation of T372 abrogates LD localization and that of S406 increases the enzymatic activity of ATGL. The C-terminus from amino acids 289–486 suppresses enzymatic activity and might interfere with ABHD5 interactions. Fat-specific protein-27 (FSP27) possibly interferes with LD localization or with ABHD5 interaction (dashed lines). For murine HSL, the N-terminus serves as a docking domain for interaction partners. Adipocyte-type fatty acid-binding protein (A-FABP) binds to amino acids 192–200, with H194 and E199 being critical for interaction. The C-terminal domain contains the catalytic domain (grey) consisting of S423, D703, and H733, and within that, a regulatory domain (light green) with five phosphorylation sites that regulate HSL localization and activity, and a putative lipid binding domain (italics, dashed line). For human MGL, the active site consists of S122, D239, and H269, and the cap domain (grey) covering the active site that stretches from amino acids 151 to 225. Putative binding partners that may regulate activity or protein expression are indicated by dashed circles (SND1, Staphylococcal nuclease and tudor domain containing 1). Amino acids of the catalytic sites are highlighted in bold. Co-regulators/amino acids that activate enzyme activity are highlighted in green; those who inhibit enzyme activity are highlighted in red color tones.

In 2004, ATGL (officially annotated as patatin-like phospholipase domain containing 2, PNPLA2; also known as desnutrin and iPLA2-zeta) was identified as a robust TAG hydrolase, leading to a paradigm shift in the understanding of intracellular TAG breakdown [13,14,15]. ATGL is expressed in most tissues with highest levels detected in white and brown adipose tissue (AT). ATGL is a potent TAG hydrolase, preferentially cleaving long-chain FAs at the *sn*-2 position of the glycerol backbone [16]. Additionally, it exhibits minor phospholipase [17], DAG transacylase [15], and retinyl ester hydrolase activities [18]. Although the three-dimensional structure of ATGL remains to be determined, sequence-based structure predictions shed some light on the enzyme’s domain organization (Figure 3). ATGL belongs to the PNPLA family, with an alpha/beta/alpha sandwich architecture and a patatin domain in the N-terminal half of the protein. This domain contains the catalytic dyad composed of Ser47 located within a GXSXG motif and Asp166 [19]. A hydrophobic amino acid region stretches from Val315 to Ile364 that is essential for the LD localization of the enzyme. The C-terminal region of ATGL also possesses a negative auto-regulatory function [20]. Phosphoproteomic studies revealed that murine ATGL has at least eight phosphorylation sites [21]. The functional relevance of two of these phosphorylation sites has been investigated in more detail. Phosphorylation at Ser406 increases the TAG hydrolase activity of ATGL [22], whereas phosphorylation at Thr372 abrogates its LD localization [21]. The respective kinases are still a matter of controversy.

Consistent with its crucial role in lipolysis and energy homeostasis, the loss of ATGL activity provokes severe metabolic perturbations in many species. ATGL-knockout (KO) mice accumulate TAGs in most tissues and die prematurely from cardiomyopathy [23]. The cardiac dysfunction also leads to a severe defect in thermogenesis in ATGL-KO mice [24]. Defective TAG utilization in ATGL-KO mice causes a shift from FA to glucose oxidation, which is associated with increased insulin sensitivity and glucose tolerance [23,25]. Resistance to high-fat diet-induced obesity in ATGL-KO mice indicates that ATGL is linked to lipid synthesis and AT growth [25]. In humans, loss of function mutations in the ATGL gene cause a rare, autosomal recessive condition called neutral lipid storage disease with myopathy (NLSD-M) [26]. The clinical manifestations of NLSD-M patients resemble the phenotype of ATGL-KO mice with respect to TAG accumulation in ectopic tissues, including peripheral leukocytes, and progressive (cardio)myopathy [5].

Over time, it became clear that ATGL and the other lipolytic enzymes do not act in solitude but instead interact with numerous protein partners within the lipolysome. Currently, more than a dozen proteins are known to bind to ATGL and affect its enzymatic properties. ATGL’s interaction partners and site of interaction are highlighted in Figure 2 and Figure 3.

### 2.1. Alpha/Beta-Hydrolase Domain Containing Protein 5 (ABHD5)

Two years after the identification of ATGL, a potent activator of its activity was discovered. ABHD5 (also annotated as comparative gene identification-58, CGI-58) binds to ATGL and stimulates its TAG and retinyl ester hydrolase activities [18,27]. ABHD5 exhibits a broad tissue expression pattern with highest abundance in white and brown AT [28]. The exact molecular mechanism by which ABHD5 activates ATGL remains elusive. However, several structural domains in both ATGL and ABHD5 have been identified to be crucial for activation. ABHD5 interacts with the patatin domain of ATGL, but the exact amino acid residues involved in the interaction are not known [19,20]. Interestingly, a truncated ATGL variant lacking 220 C-terminal amino acids (where amino acids 1 to 289 are still present) shows an increased binding affinity to ABHD5 and a higher TAG hydrolase activity, indicating that the C-terminus of ATGL interferes with its interaction with ABHD5 [20]. Within ABHD5, the 30 N-terminal residues harbor a lipophilic tryptophan-rich stretch, which is crucial for both LD localization and ATGL activation [29]. Recent data indicate that Arg299 and Gly328 located near the C-terminus of ABHD5 are also important for activating ATGL-dependent lipolysis, probably by creating perturbations in the LD membrane and facilitating ATGL’s access to TAGs [30].

Several genetic models substantiate the role of ABHD5 as a potent regulator of ATGL and intracellular TAG catabolism. Consistent with reduced TAG hydrolase activities, ABHD5-KO mice show significantly increased carcass neutral lipid content with severe hepatic steatosis. In contrast to ATGL-KO mice, however, these mice die perinatally due to a severe skin barrier defect with transepidermal water loss [6]. This observation supported the notion that ABHD5 also exhibits ATGL-independent functions that promote acylceramide synthesis and the formation of the epidermal corneocyte lipid envelope [31]. In humans, mutations in the gene encoding *ABHD5* are causally linked with Chanarin Dorfmann Syndrome, also designated as neutral lipid storage disease with ichthyosis (NLSD-I) [26,32]. This autosomal recessive disease has been reported in more than 100 individuals, mostly from the Mediterranean. Similar to ABHD5-KO mice, patients accumulate neutral lipids in ectopic tissues and suffer from severe ichthyosis due to a skin barrier defect [33].

Recently, two studies demonstrated that ABHD5 also binds and activates PNPLA1, a close relative of ATGL, predominantly expressed in skin keratinocytes. ABHD5 may activate a transacylase activity of PNPLA1 that could be essential for the formation of epidermal ω-O-acylceramides [34,35]. Another identified ABHD5 interaction partner is PNPLA3 (also designated as adiponutrin) [36]. This is not surprising, given the high sequence homology between ATGL and PNPLA3, especially within the conserved patatin domain responsible for the interaction between ATGL and ABHD5 [19]. PNPLA3 was shown to sequester ABHD5, thereby limiting its availability for ATGL activation and, thus, lowering ATGL-dependent TAG hydrolysis, particularly in hepatocytes [36]. ABHD5 is required for PNPLA3 to localize to LDs. In contrast to the wild-type protein, PNPLA3 (I148M) mutant accumulates on the LD, sequesters ABHD5, and causes severe hepatic steatosis in mice and humans [37,38,39].

ABHD5 also interacts with various members of the fatty acid-binding protein (FABP) family, including adipocyte-type (A-FABP), heart-type, liver-type, intestinal-type, and epidermal-type FABP [40]. FABPs constitute a multi-protein family of nine members that share the ability to bind FAs and other hydrophobic ligands. Each member has its own characteristic tissue distribution, with A-FABP being mainly expressed in AT, macrophages, and dendritic cells [41]. In general, FABPs function as lipid chaperones, escorting lipids and dictating their biological functions. In particular, the interaction of ABHD5 and A-FABP further stimulates ABHD5-mediated ATGL activity and participates in the nuclear import of FAs to regulate the activity of nuclear receptors. However, a direct interaction of ATGL and FABP was not observed [40].

### 2.2. Perilipins (PLINs)

Both ATGL and ABHD5 bind to PLINs on the surface of LDs. In mammals, there are five perilipin genes encoding five major PLIN proteins (named in the order of their discovery as PLIN 1–5) with strong N-terminal sequence homology [42]. The N-terminal region of each PLIN harbors two characteristic motifs. There is an approximately 100 amino acid PAT domain (derived from perilipin, ADRP, and TIP47; the synonyms of PLIN 1, 2, and 3, respectively) localized at the N-terminus. The PAT domain is followed by a sequence of 11-mer repeats predicted to fold into an LD-anchoring, amphipathic helix. The C-terminus varies significantly between the PLIN family members [43]. Moreover, there are marked differences in their tissue distribution patterns, properties of binding to lipolytic proteins, and, thus, physiological roles.

PLIN1 is mainly expressed in the adipocytes of brown and white AT, where it coats mature, mostly unilocular, LDs. PLIN1 harbors a C-terminal binding site for ABHD5, spanning amino acid residues 382–429 [28]. Under basal conditions, PLIN1 binds and sequesters ABHD5, thereby limiting the availability of ABHD5 to interact with and stimulate ATGL and, thus, preventing accelerated lipolysis. Consistently, PLIN1-KO mice [44] and humans with frameshift mutations altering the C-terminus of PLIN1 [45,46] show unrestricted basal lipolysis and suffer from lipodystrophy.

This interaction network changes profoundly upon the stimulation of lipolysis. In times of increased energy demand (i.e., upon fasting or exercise), catecholamines bind to G-protein coupled β-adrenergic receptors on the surface of adipocytes [42]. As a result, the Gs subunit dissociates from the receptor to bind and activate adenylate cyclase, converting ATP to cAMP. Elevated cAMP concentrations activate the catalytic subunits of protein kinase A (PKA, also designated as cAMP-dependent protein kinase) by releasing the regulatory subunits from the tetrameric kinase. PKA then phosphorylates many proteins of the lipolysome. PLIN1 carries at least six serine residues within PKA consensus sequences [47], while ABHD5 has one PKA consensus sequence [48]. The phosphorylation of Ser492 and Ser517 of PLIN1 [49] and Ser239 of ABHD5 [48] is required to fully release ABHD5 from PLIN1, enabling ABHD5-mediated ATGL activation. Interestingly, ATGL was also observed to translocate to PLIN1-coated LDs following PKA activation, even though a direct interaction between ATGL and PLIN1 has been excluded [50]. The mechanism of this translocation still awaits clarification.

PLIN2 is ubiquitously expressed and represents the predominant LD-associated PLIN isoform in tissues that do not express PLIN1 or PLIN5 [42]. PLIN2 was reported to interact with both ATGL [51] and ABHD5 [52]. Nevertheless, PLIN2 only moderately controls cytosolic lipolysis for several reasons. First, PLIN2 is not phosphorylated by PKA [42] and, hence, does not respond to β-adrenergic stimulation. Second, due to structural differences in the C-terminal region, PLIN2 is less effective than PLIN1 in sequestering ABHD5 and preventing it from interacting with ATGL [53]. Thus, PLIN2 does not restrict lipolysis as efficiently as PLIN1, consistent with the relatively mild phenotype of PLIN2-KO mice on a chow diet [54].

PLIN3 also shows a broad tissue distribution but localizes more to nascent LDs, being replaced by PLIN2 when the LD size increases [42]. PLIN3 can be phosphorylated by PKA and also interacts with both ATGL and ABHD5 [51], again to a lesser extent than PLIN1. PLIN3-KO mice have unaltered AT mass but show beiging of white AT depots, with the occurrence of uncoupling protein 1 expression, multilocular adipocytes, and increased cold tolerance [55]. The knockdown of PLIN3 in AML-12 hepatoma cells similarly increases the fraction of small LDs without affecting overall TAG content [56]. Interestingly, FA and glycerol release from the inguinal white AT of PLIN3-KO mice is only increased when the mice are housed at 4 °C but not at 30 °C [55]. This might indicate that PLIN3 controls stimulated lipolysis more strongly than basal lipolysis. Whether and how its interactions with ATGL and ABHD5 are involved therein still need to be addressed.

PLIN4 is mainly expressed in white AT, and to lesser degree in skeletal muscle and the heart [57]. It shows a strong structural divergence from the other PLIN isoforms due to (i) an extended sequence of imperfect 11-mer repeats giving rise to an amphipathic helix of exceptional length [58] and (ii) the absence of the PAT domain [59]. PLIN4 preferentially localizes to LDs enriched in cholesteryl esters (CEs) rather than TAGs [42]. Due to its strong interaction with neutral lipids, yet weak interaction with lipid bilayers in vitro, PLIN4 is believed to directly contact the LD core and substitute the phospholipid coating [58]. Possible interactions between PLIN4 and ATGL or ABHD5 have not been investigated to our knowledge. PLIN4-KO mice have unaltered AT morphology and function, yet exhibit reduced cardiac TAG content [60]. However, PLIN5 expression is concomitantly reduced in these hearts [60], making it difficult to dissect the individual role of PLIN4 in controlling the lipolysome.

PLIN5 is mainly present in tissues with high oxidative capacity including skeletal muscle, heart, brown AT, and liver [42]. PLIN5-KO mice exhibit reduced TAG content in these tissues compared to their wild-type littermates [61,62,63,64]. Equivalent to PLIN1 in white AT, PLIN5 strongly restricts LD turnover in oxidative tissues. However, the mechanism appears to be different than it is for PLIN1. Granneman et al. [50] reported that the C-terminal half of PLIN5 carries overlapping binding sites for ATGL and ABHD5 spanning amino acids 200–453. Fluorescence imaging revealed that PLIN5 overexpression recruits both ATGL and ABHD5 to the LD surface. However, the interaction between PLIN5 and ATGL or ABHD5 is mutually exclusive, meaning that one molecule of PLIN5 can either bind ATGL or ABHD5 but not both proteins simultaneously [50]. Therefore, the authors concluded that ATGL and ABHD5 can no longer interact with each other when they are recruited to PLIN5-coated LDs; thus, basal lipolysis is attenuated.

PLIN5 carries at least one serine residue (Ser155) within a PKA consensus motif and additional phosphorylation sites for other kinases [65,66]. Pollak et al. [65] demonstrated that mutating Ser155 to an alanine residue strongly attenuates stimulated lipolysis in the heart, indicating that PKA-mediated PLIN5 phosphorylation promotes lipolysis. Thus, it is tempting to speculate that PLIN5, similarly to PLIN1, releases ABHD5 upon phosphorylation, but experimental evidence is still missing. The characteristics of phosphorylated PLIN5’s binding to ATGL (and HSL) need to be investigated.

### 2.3. G0/G1 Switch Gene 2 (G0S2)

In 2010, the hypoxia-inducible protein G0S2 was identified as an ATGL inhibitor [67]. G0S2 is mainly expressed in AT and its expression increases upon adipogenesis [68]. The mechanism of ATGL inhibition requires the interaction of the hydrophobic linker domain of G0S2 with the patatin domain of ATGL [67]. The inhibitory function of G0S2 on ATGL was confirmed using transgenic animal models overexpressing G0S2 in adipocytes or cardiomyocytes causing TAG accumulation in respective cells [69,70]. By contrast, the G0S2-KO phenotype is relatively mild, probably due to the low G0S2 transcript levels found in most tissues. The highest G0S2 mRNA levels are observed in AT and, accordingly, G0S2-KO mice exhibit increased lipolysis, decreased AT mass, and resistance to diet-induced obesity when fed a high-fat diet [7,8].

Similarly to ABHD5, G0S2 also exerts ATGL-independent pleiotropic functions. It specifically binds to the oxidative phosphorylation complex V (ATP synthase) to increase ATP production rates, a process that is hypothesized to circumvent energy depletion, especially under hypoxic conditions [71]. In bone marrow hematopoietic cells, G0S2 was shown to interact with nucleolin (NCL) to retain this typically nucleolar RNA-binding protein in the cytosol [72]. As a consequence, NCL cannot exert its otherwise pro-proliferative actions, and cells stay in a quiescent state. Another G0S2 interaction partner is B-Cell CLL/Lymphoma 2 (Bcl2), an anti-apoptotic protein [73]. Whether and how oxidative phosphorylation, cell cycle progression, and apoptosis “crosstalk” with lipolysis via G0S2 remains to be elucidated.

### 2.4. Hypoxia-Inducible LD-Associated Protein (HILPDA)

Recently, HILPDA (also designated as hypoxia-inducible gene-2, HIG-2) was identified as a second ATGL inhibitor. HILPDA is structurally similar to G0S2, is ubiquitously expressed upon hypoxia, and localizes to the LD [74,75,76]. HILPDA directly binds and inhibits ATGL in vitro [75,77]. The contact region between HILPDA and ATGL was mapped to the hydrophobic N-terminus of HILPDA and the patatin domain of ATGL [77]. Consistently, deleting the amino acid region Leu7 to Gly11 in HILPDA completely abolishes its binding to ATGL [75]. Kinetic studies determining the IC_50_ values revealed that HILPDA is, by two orders of magnitude, less potent as an ATGL inhibitor than G0S2 [77], raising doubts as to whether it is a direct regulator of lipolysis [78]. While HILPDA overexpression or gene deletion affected cellular TAG content in various cancer cells and hepatocytes [74,75,76], no clear impact on TAG hydrolysis was observed in adipocytes [77].

### 2.5. Fat-Specific Protein-27 (FSP-27)

Another inhibitory lipolysome member is FSP-27 (also designated as cell death-inducing DNA fragment factor 40/45-like effector C, CIDEC). FSP-27 is found exclusively in white and brown AT, and its mRNA is upregulated during adipogenesis [79]. The absence of FSP-27 in mice and humans causes lipodystrophy paralleled by the occurrence of atypical, multilocular white adipocytes, and higher lipolysis rates [9,10]. Conversely, FSP-27 overexpression is associated with the formation of supersized LDs and neutral lipid accumulation in various cell types [79].

How FSP-27 inhibits lipolysis is not fully understood. Growing evidence suggests that FSP-27 acts on multiple levels to control LD size and TAG content. Firstly, FSP-27 was demonstrated to be enriched at LD contact sites in 3T3-L1 adipocytes, where it mediates unidirectional neutral lipid transfer from smaller to larger LDs [80]. On a molecular level, this function is regulated by a polybasic linker domain electrostatically interacting with phospholipids [81]. The FSP-27-elicited production of large, unilocular LDs involves the interaction of FSP-27 with PLIN1 [82,83]. Secondly, FSP-27 interacts with ATGL and inhibits ATGL-mediated glycerol release [84]. Unlike G0S2 and HILPDA, however, FSP-27 does not inhibit ATGL’s in vitro TAG hydrolase activity [85], suggesting that FSP-27 limits ATGL’s access to LDs or interferes with its interaction with ABHD5. FSP-27′s inhibitory impact on lipolysis can also explain the pro-lipolytic function of growth hormone (GH). GH interferes with peroxisome proliferator-activated receptor (PPAR) gamma signaling by promoting its nuclear export via PPAR gamma Ser273 phosphorylation, which, as a consequence, downregulates FSP-27 and activates lipolysis [86].

### 2.6. Pigment Epithelium-Derived Factor (PEDF)

Since its discovery as a glycoprotein secreted from cultured pigment epithelial cells, numerous follow-up studies have proved PEDF to be involved in a wide range of biological processes including angiogenesis, neuroprotection, tumor growth, and metastasis. PEDF is ubiquitously expressed and, consistent with its intrinsic leader sequence responsible for secretion, abundantly found in the plasma [87]. Interestingly, AT is a major source of circulating PEDF, and PEDF plasma concentrations are elevated in obese individuals [88].

Searching for potential PEDF receptors, Notari et al. [17] identified ATGL as a high-affinity, membrane-associated PEDF binding partner using a combination of biochemical and biophysical methods. In vitro hydrolase activity assays revealed that recombinantly expressed ATGL exhibits phospholipase activity, which can be stimulated by the addition of purified PEDF. According to these findings, extracellular PEDF would be able to exert its functions via activating membrane-bound ATGL. However, to our knowledge, the latter study remains the only one to show that ATGL localizes to the plasma membrane, while a large body of evidence suggests that ATGL primarily localizes to the cytoplasm and LDs. LD-associated ATGL also interacts with PEDF after its cellular uptake [89,90,91]. Several observations indicate that PEDF activates ATGL-mediated TAG hydrolysis, at least in the liver, cardiac muscle, and AT: (i) A genetic deficiency of PEDF causes hepatic TAG accumulation. This phenotype can be reversed by treating PEDF-KO hepatocytes with recombinant PEDF, unless ATGL is concomitantly inhibited [90]. PEDF is also downregulated in diet-induced hepatosteatosis [89]. (ii) Recombinant PEDF increases basal lipolysis in wild-type but not ATGL-KO AT explants [91]. (iii) Recombinant PEDF decreases the TAG content of cardiomyocytes solely in the presence of ATGL [92]. Data on the interaction regions are scarce. Mutational studies have been performed to map the regions involved in the ATGL/PEDF interaction. PEDF co-immunoprecipitates with human ATGL lacking amino acids 1–267 but not with human ATGL lacking amino acids 268–504 [89], implying that PEDF binds to the C-terminal half of ATGL. Consistent with the finding that G0S2 binds to the patatin domain of ATGL [67] located within its N-terminus, the authors observed that ATGL can simultaneously bind PEDF and G0S2. A 44-mer peptide derived from PEDF (Val78-Thr121) decreases cardiomyocyte TAG content to the same extent as full-length PEDF [92], suggesting that ATGL binding involves amino acid residues located between Val78 and Thr121 of PEDF. However, co-immunoprecipitation studies using different mutants are needed to prove this hypothesis.

In conclusion, PEDF likely acts as a pro-lipolytic factor by activating ATGL, although mechanistic details remain elusive. The functional contribution of extracellular PEDF provoking receptor-mediated signaling events needs to be revisited.

### 2.7. Ubiquitin Regulatory X Domain-Containing Protein 8 (UBXD8)

Olzman et al. [93] reported an interaction between ATGL and UBXD8. Mechanistically, the authors propose that UBXD8 or the segregase activity of its interaction partner p97 subunit/valosin containing protein (p97/VCP) disassembles the ATGL/ABHD5 complex. Accordingly, the overexpression of UBXD8 increases LD size and number in HeLa cells. Since UBXD8 is not found in adipocytes, it may be more relevant for the inhibition of lipolysis in non-adipose tissues. However, recent data from UBXD8-KO mice provided no evidence for a “hyperlipolytic” state [94]. By contrast, UBXD8-KO mice developed liver steatosis when fed a high-fat diet. Further gain- or loss-of-function studies are required to estimate the physiological relevance of the UBXD8/ATGL interaction.

### 2.8. Golgi Brefeldin a Resistance Factor 1 (GBF1)

GBF1 is another lipolysome member that directly interacts with ATGL via several contact regions [95]. GBF1 acts as a guanine nucleotide exchange factor for ADP-ribosylation factor 1 (ARF1), which in turn affects the coatomer protein I (COPI). The GBF1/ARF1/COPI protein ensemble mediates retrograde protein transport from the Golgi network to the ER, and cargo export from the ER [96,97]. This process apparently affects ATGL targeting to the LD surface, since the inactivation of the protein ensemble by Brefeldin A or GBF1/COPI silencing abrogates ATGL’s LD association upon oleic acid treatment [95]. In both cases, ATGL accumulates in the membrane fraction, more precisely in ER exit sites. Accordingly, GBF1/COPI silencing also results in oversized LDs in HeLa cells and *Drosophila melanogaster* [98,99,100]. Whether GBF1 affects basal and/or activated lipolysis needs to be clarified.

### 2.9. 14-3-3

14-3-3 proteins constitute a highly conserved regulatory protein family of seven isoforms affecting a large variety of cellular processes [101,102,103]. More than 300 different 14-3-3 interaction partners have been reported, most of which contain consensus sequences with phosphorylated serine or threonine residues. ATGL is one of these interaction partners [22]. A non-phosphorylatable ATGL mutant failed to bind 14-3-3 in vitro and exhibited decreased in vitro TAG hydrolase activity, suggesting that ATGL might be stabilized in response to 5’ adenosine monophosphate-activated protein kinase (AMPK) phosphorylation and 14-3-3 binding. The *Caenorhabditis elegans* ATGL homolog was also shown to be phosphorylated by AMPK, generating a 14-3-3 binding site, which, in contrast to the other study, sequesters ATGL away from the LD surface and decreases its activity [104]. Currently, no consistent model exists for the role of 14-3-3 in the regulation of ATGL localization, stability, or activity.

### 2.10. Peroxisome Biogenesis Factor 5 (PEX5)

Proteins of the PEX family are essential for peroxisome biogenesis and the sequestering of peroxisomal proteins from the cytoplasm to nascent peroxisomes [105]. Among those, PEX5, or peroxisome target sequence 1 receptor protein (PTS1R), which occurs predominantly in the cytoplasm, shuttles PTS1 binding peroxisomal proteins from the cytoplasm to the peroxisome [105,106]. Recently, PEX5 has been reported to regulate fasting-induced lipolysis by direct interaction with ATGL [107]. Using *Caenorhabditis elegans*, 3T3-L1 adipocytes, and mice, the authors demonstrate that peroxisome-LD interactions are enhanced upon fasting and necessary for fully activated fasting-mediated lipolysis. Apparently, fasting induced the Kinesin Family Member C3 (KIFC3)-dependent movement of peroxisomes towards LDs and enabled ATGL co-localization to peroxisome-LD contact points. The genetic and pharmacological inhibition of peroxisome movement prevents ATGL translocation and diminishes fasting-induced lipolysis. RNAi screening in *Caenorhabditis elegans* revealed PEX5 to be responsible for ATGL recruitment, and further co-immunoprecipitation and imaging studies proved the direct interaction of PEX5 and ATGL. In line with these findings, PEX5-KO mice showed reduced ATGL LD localization and reduced fasting-induced lipolysis. The simultaneous knock down of PEX5 and ABHD5 was able to lower lipolytic activity in 3T3-L1 cells, but the co-suppression of ATGL and ABHD5 yielded similar results compared to the single knock-down of ATGL or PEX5. Based on these findings and the fact that ABHD5 suppression did not alter ATGL localization on LDs, the authors concluded that PEX5 acts independently of ABHD5 on lipolysis. Although this study elegantly described a novel regulatory mechanism of lipolysis, several aspects such as the involvement of ABHD5, other LD components, and lipases have to be clarified.

## 3. HSL and Its Interaction Partners

HSL was initially described in 1964 to be an AT lipid hydrolase induced by fasting and hormones [108,109,110]. Although its expression is highest in white and brown AT, it is also detectable in many other tissues and cell types including cardiac and skeletal muscle, liver, kidney, pancreas, and steroidogenic tissues [111,112,113]. HSL exhibits a broad substrate spectrum, hydrolyzing TAGs, DAGs, MAGs [114], retinyl esters, CEs [115,116], steroid hormone FA esters [117], and even water soluble, short-chain FA ester substrates [118]. HSL shows the highest activity against sn-1,3 DAGs followed by CEs and TAGs [16,112].

HSL-KO mice accumulate DAGs in AT, muscle, and the testis [119] Unlike ATGL-KO mice, HSL-KO mice do not develop cardiomyopathy or cold intolerance [119,120], but male HSL-KO mice are sterile [120]. Remarkably, HSL-KO mice develop partial lipodystrophy, particular upon advanced age, which is associated with hepatosteatosis [121]. Similarly to mice, humans lacking HSL protein also accumulate DAGs in white AT and develop lipodystrophy and liver steatosis [122]. The mechanistic role of HSL in adipogenesis, AT growth, and degradation is currently poorly understood and deserves major attention.

Although HSL has been intensively studied, its crystal structure has not yet been solved. According to the current domain structure model, the 82 kDa enzyme contains four functionally relevant domains: (1) the N-terminal domain, which is essential for HSL dimerization and serves as docking domain for protein-protein interactions [123]; (2) the C-terminal domain harboring the catalytic triad within a typical alpha/beta-hydrolase fold [124,125,126]; (3) the regulatory domain containing five known phosphorylation sites (mouse HSL: Ser563, Ser659, and Ser660 are phosphorylated by PKA, Ser565 by AMPK, and Ser600 by extracellular signal-regulated kinase (ERK); human HSL: Ser552, Ser649, and Ser650 are phosphorylated by PKA, Ser554 by AMPK, and Ser589 by ERK [127,128,129,130,131,132]), which regulate the localization and activity of HSL [133]; and (4) the putative lipid-binding domain [134].

Just like ATGL, HSL also requires protein-binding partners that regulate enzyme localization and activity. Several of these are shared by both lipases. (Putative) domain structures, phosphorylation sites, and interaction partners of murine HSL are shown in Figure 2 and Figure 3.

### 3.1. FABPs

As described above, virtually all members of the FABP family interact with ABHD5 to regulate ATGL-mediated lipolysis. Two members, A-FABP and epidermal-type-FABP, also bind to HSL [135,136,137,138]. Apparently, HSL is more selective than ABHD5 for FABP binding because liver-type- and intestinal-type-FABP do not interact with the lipase [135,139]. The overexpression of fluorescently tagged HSL and A-FABP in adipocyte-like cells demonstrated that the A-FABP/HSL complex already exists under basal conditions in the cytosol and translocates to the LD surface in response to the hormonal stimulation of lipolysis [135].

A-FABP directly interacts with HSL at its N-terminal region from amino acids 192 to 200 [140]. In this sequence, two specific residues (His194 and Glu199) have been identified that determine the binding of HSL to A-FABP (Figure 3). Additionally, Smith et al. [137] demonstrated that the interaction is dependent on HSL phosphorylation. Mouse HSL with mutations at two PKA phosphorylation sites, Ser659 and Ser660, or at the AMPK phosphorylation site Ser565 failed to interact with A-FABP in living cells. However, incoherent reports exist on the functional consequences of the A-FABP/HSL interaction. Initial studies showed that either the co-incubation of in vitro translated HSL with purified A-FABP or the co-expression of HSL and A-FABP in CHO-cells increases the hydrolytic activity of HSL against CEs [136]. In contrast, A-FABP-KO mice exhibit decreased lipolysis in vivo and in situ [141]. Therefore, it was assumed that A-FABP prevents HSL from product inhibition by sequestering the produced FAs. Consistently, A-FABP did not alter HSL’s activity against p-nitrophenyl butyrate, which was explained by the fact that FABPs do not bind the released reaction product, butyric acid [139]. Interestingly, liver-type- and intestinal-type FABP, despite not interacting with HSL, also stimulated its CE hydrolase activity [139], leading to the conclusion that physical association is not necessary for activation. However, Shen et al. [140] reported that both wild-type A-FABP and FA-binding defective A-FABP (A-FABP^R126L^) bind to and stimulate HSL activity in vitro. These data support a scenario where A-FABP binding to HSL leads to conformational changes in the lipase, causing enzyme activation. But this interaction was not present when another FA binding defective mutant of A-FABP (A-FABP^R126Q^) was used [137]. Consistently, the presence of oleate was a prerequisite to the detection of A-FABP/HSL complexes in isothermal titration calorimetry experiments [139]. These observations led to the hypothesis that solely ligand-bound A-FABP binds to HSL to inhibit its hydrolytic activity in a negative feedback loop.

### 3.2. PLINs

The interaction of PLIN1 with HSL contributes significantly to HSL regulation upon hormonal stimulation of lipolysis. The binding of catecholamines to β-adrenergic receptors leads to the activation of adenylate cyclase and, hence, the elevation of intracellular cAMP levels. This, in turn, activates PKA, which directly phosphorylates HSL at the residues Ser563, Ser659, and Ser660. However, the modestly increased activity of phosphorylated HSL in vitro could not explain why catecholamines stimulate lipolysis up to 100-fold in adipocytes [142]. This apparent discrepancy was explained by follow-up studies showing that PKA also phosphorylates PLIN1 at multiple serine sites (rodent PLIN1 has six residues: Ser81, Ser222, Ser276, Ser433, Ser492, and Ser517; human PLIN1 has five residues: Ser81, Ser276, Ser433, Ser497, and Ser522), which causes conformational changes to strengthen its interaction with HSL. This fundamental process recruits HSL to the LD surface and enhances its substrate access for full lipolytic stimulation [143,144,145,146,147]. In accordance with this model, Wang et al. [148] demonstrated that the direct interaction between PLIN1 and HSL on LDs significantly increases following PKA stimulation of cells. Although the interaction of HSL and PLIN1 was verified repeatedly, the binding site(s) of HSL to PLIN1 is (are) still debatable [148,149]. Wang et al. [148] showed an interaction of the lipase within the highly conserved N-terminal PAT domain, while Shen et al. [149] identified HSL binding sites on the N-terminus outside of the PAT region and on the C-terminus. These sites not only overlap with sequences that have been implicated in LD barrier function [150], but also with sequences that interact with ABHD5 [28,52], suggesting that the interaction of HSL with PLIN1 facilitates multiple functions including the recruitment of HSL to LDs as well as the regulation of lipolysis [149].

Wang et al. [148] further demonstrated that HSL also interacts with PLIN2, 3, and 5 (PLIN4 was not investigated) via their N-terminal PAT domains to regulate the localization of HSL to LDs in non-adipose cells. In contrast to PLIN1, PLIN2, 3, and 5 already interact with HSL under basal conditions, and their binding to the lipase is either reduced (PLIN2) or unchanged (PLIN3, 5) upon PKA stimulation, suggesting the involvement of additional regulatory mechanisms to control lipolysis [148].

### 3.3. Vimentin (VIM)

VIM is an intermediate filament protein that is part of the cytoskeletal network. Additionally, it is located on LDs under both basal and lipolysis-stimulated conditions [151]. VIM binds to HSL, and this interaction is controlled by hormones. PKA stimulates, while insulin inhibits, VIM binding to HSL [152]. Notably, VIM was also found to interact with β_3_-adrenergic receptors to transduce the lipolytic signal via the ERK pathway in response to catecholamines. The pharmacological disruption of VIM filaments blocks ERK activation and reduces CL 316,243 stimulated lipolysis in adipocytes, but not forskolin-stimulated lipolysis, which is mainly mediated by PKA activation [153]. Disruption of the endogenous VIM intermediate filament network impairs LD formation and TAG accumulation in 3T3-L1 adipocytes [154]. Consistently, VIM-KO mice exhibit smaller adipocytes [152]. Furthermore, adipocytes isolated from VIM-KO mice show both reduced HSL translocation to the LDs and a decreased lipolytic response to isoproterenol [152]. These data suggest a close functional relationship between lipolysis and the cytoskeleton, which requires mechanistic validation.

### 3.4. Cavin-1

Another HSL interaction partner is cavin-1 (also designated as polymerase I and transcript release factor, PTRF) which is a major structural component of caveolae [155]. Plasma membrane invaginations in the form of caveolae are found in most mammalian cell types and are particularly abundant in white adipocytes. Caveolae are believed to be involved in the uptake and storage of FAs and their release [156]. A deficiency of cavin-1 in mice or humans results in dysregulated lipid metabolism, including the impaired hydrolysis and synthesis of TAGs, leading to lipodystrophy [157,158,159,160].

Proteins involved in lipolysis such as HSL and PLIN1 are also present in caveolae, maintaining a coordinated TAG turnover [161]. Hence, Aboulaich and colleagues [162] demonstrated a direct interaction of cavin-1 with HSL showing that insulin treatment triggers cavin-1 and HSL co-migration from the plasma membrane to the cytosol in primary human adipocytes. A recent study further showed that acetylation of cavin-1 promotes interaction with HSL and recruits the lipase to the caveolae to increase lipolysis [163]. Besides these direct effects of cavin-1 to control lipolysis via HSL translocation, cavin-1 might further modulate HSL-mediated lipolytic activity indirectly via the regulation of PLIN1 phosphorylation. As previously shown, PLIN1 phosphorylation is required for HSL activity (see above) [145]. Accordingly, the phosphorylation of PLIN1 is blunted in cavin-1-KO mice, resulting in impaired stimulated lipolysis [160], which might be partly due to the reduced lipolytic activity of HSL, suggesting that cavin-1 is a critical regulator of HSL activity at caveolae.

### 3.5. Carbohydrate Response Element Binding Protein (ChREBP)

ChREBP is a glucose-activated transcription factor that regulates de novo lipogenesis (DNL) in multiple tissues including the liver and AT [164]. In humans, the expression of ChREBP and adipose DNL are positively associated with insulin sensitivity [165,166]. Recently, an exciting study demonstrated a direct interaction between HSL and ChREBP regulating insulin signaling in white adipocytes [167]. Thereby, HSL retains ChREBP in the cytoplasm, preventing its nuclear translocation and subsequent transcriptional induction of target genes such as the FA elongase ELOVL6, an enzyme catalyzing the elongation of palmitic acid to stearic acid, which can be further desaturated into oleic acid. By contrast, the partial inhibition of HSL, which was shown to improve insulin sensitivity in murine AT and human adipocytes [167,168], increases ELOVL6 expression. As a consequence, increased amounts of oleic acid are incorporated into phospholipids. This change in phospholipid composition alters plasma membrane properties and improves insulin signal transduction.

Morigny et al. [167] further showed that an enzymatically inactive form of HSL represses ChREBP activity as well, indicating that the protein-protein interaction depends on the presence of HSL but not on its lipolytic activity. However, it is still elusive as to which part of HSL binds to ChREBP and whether the phosphorylation of the lipase modifies their interaction.

### 3.6. Microtubule-Associated Proteins 1A/1B Light Chain 3B (LC3)

LC3 is a crucial component of autophagosomes. Besides its pro-autophagic activity, LC3 has been recently implicated in regulating lipolysis via modulating ATGL and HSL activity [169]. Both lipases contain multiple LC3-interacting region (LIR) motifs in their primary sequences and co-localize with LC3 in response to cold exposure as well as in response to rapamycin-induced autophagy with LDs in brown AT and the liver. In addition, cold exposure also enriches these lipases on the cytoplasmic site of LC3/autophagosomes. Using co-immunoprecipitation, Martinez-Lopez et al. [169] confirmed a direct interaction of HSL with LC3 that increases by inducing autophagy. Although not investigated, LC3 most likely binds to the N-terminal region of HSL, since five of the seven LIR motifs are located at the N-terminus. Furthermore, mutating a single LIR motif of ATGL resulted in the mislocalization of ATGL and neutral lipid accumulation in an adipocyte cell line, suggesting that the LC3-ATGL interaction controls the recruitment of ATGL to LDs and, consequently, its lipolytic activity [169]. Together, the interaction of LC3 with ATGL and HSL might coordinate lipolysis and (lipo-)autophagy.

### 3.7. Steroidogenic Acute Regulatory Protein (StAR)

StAR is a critical regulator of steroid biosynthesis by facilitating the intracellular transfer of cholesterol from the outer to the inner mitochondrial membrane [170]. HSL, as a main CE hydrolase, provides cholesterol for steroidogenesis [120]. Hence, Shen et al. [171] demonstrated a direct interaction of HSL with the N-terminal and central regions of StAR. Consequently, HSL-mediated CE hydrolase activity increases in the presence of StAR, suggesting that their interaction results in conformational changes of the lipase that either enable efficient substrate access or reduce product inhibition. Furthermore, the overexpression of HSL in adrenal cells increases mitochondrial cholesterol content when StAR expression is induced, implying that both proteins form a functional complex to promote the release and transfer of cholesterol from cytoplasmic LDs to mitochondria for steroidogenesis.

## 4. MGL and Its Interaction Partners

MGL is the third lipase within the lipolysome hydrolyzing MAGs, thereby releasing glycerol and a FA. It belongs to the alpha/beta hydrolase protein family, exhibiting a typical alpha/beta-hydrolase fold with a GXSXG motif, and a Ser-Asp-His catalytic triad [172]. In 2010, the three-dimensional structure of human MGL was solved by X-ray diffraction by two independent groups [173,174]. The protein has a cap domain that covers the structurally conserved β-sheet and the active site that resides at the end of an unusually large hydrophobic tunnel. Additionally, human MGL provides an exit hole for the release of glycerol after hydrolysis that is located at the level of the catalytic triad. MGL is ubiquitously expressed among tissues, with slightly different molecular sizes [175]. The enzyme exhibits high hydrolytic activity against MAGs [176]; however, it was also shown to hydrolyze prostaglandin glycerol esters [177] and FA ethyl esters [178].

MGL-KO mice accumulate different MAG species in various tissues and are resistant to diet-induced obesity and protected from the development of liver steatosis [179,180,181]. In addition to its role within the lipolytic cascade, MGL is particularly important in the endocannabinoid system. It degrades 2-arachidonoyl glycerol (2-AG), the most abundant endogenous agonist of cannabinoid receptors (CBRs) [182]. The accumulation of 2-AG in MGL-KO mice provokes massive CBR desensitization and resistance to exogenous CBR agonists [183,184,185].

In contrast to the large number of published interaction partners of ATGL and HSL, only a few reports have addressed the regulation of MGL activity or expression by protein-protein interactions, but none of them have investigated a potential regulatory role in lipolysis. Based on automated yeast two hybrid interaction mating, chromogranin B (CHGB), a glycoprotein involved in vesicle sorting [186], was proposed to be a putative MGL binding partner [187]. However, functional analysis of this interaction is still elusive.

More recently, a negative correlation of Staphylococcal nuclease and tudor domain containing 1 (SND1) with MGL has been found in cancer cells [188], implying that SND1 and MGL influence each other’s expression. SND1 is a multifactorial protein acting as an oncogene in multiple cancers [189]. MGL was also shown to be involved in carcinogenesis by either promoting tumorigenesis or functioning as a tumor suppressor, depending on the tumor type [190,191]. The interaction of SND1 with MGL results in the ubiquitination and subsequent proteasomal degradation of the lipase, which eventually promotes the tumorigenic proliferation of hepatocellular carcinoma [188].

Another recent study demonstrated that MGL enhances prostate cancer metastasis in vitro and in vivo, dependent on epidermal FABP. Overexpression of MGL significantly increased PPAR gamma activity to promote a metastatic phenotype, while epidermal FABP knockdown attenuated PPAR gamma even in cells overexpressing MGL. However, functional studies to investigate whether direct protein-protein interaction is needed for this process have not been carried out [192]. Interestingly, other studies demonstrated that HSL and ABHD5 interact only with distinct FABPs. Therefore, it would be interesting to investigate whether one or more FABPs interact with MGL to alter its hydrolytic activity. An overview of human MGL’s domain structure and putative interaction partners is shown in Figure 2 and Figure 3.

## 5. Conclusions and Outlook

Protein-protein interactions are of fundamental importance to the integrity of biological networks. Hence, viewing intracellular lipolysis simply as three lipases acting sequentially to hydrolyze TAGs is far too simplistic. As summarized in this review, these lipases interact with a broad set of regulatory, accessory, and scaffolding proteins. Many of these proteins (e.g., FABPs, PLINs, and LC3) were reported to either directly or indirectly interact with both ATGL and HSL, suggesting that the lipolysome may in fact act as a macromolecular complex (Figure 2). However, the temporal and spatial dynamics of these interactions remain to be determined. Other interactions (e.g., those of ATGL/HSL with UBXD-8, cavin-1, or StAR) and their physiological consequences still need to be validated by complementary methods in order to obtain a more profound and reliable picture of the lipolysome.

To date, a high number of interaction partners have been described for ATGL and HSL, but only a few have been described for MGL. Since ATGL is the rate limiting lipase in TAG hydrolysis, it could be assumed that tight regulation of the first step of lipolysis is probably more efficient in fine-tuning lipolysis under various physiological conditions, which has attracted many studies on this specific lipase over the last decade.

In addition to such studies on the physiological role of lipolysis and its complex regulation, a quantitative description of the intricate reaction processes of TAG hydrolysis is of utmost importance. Recent advances in the biochemical characterization of the lipolytic pathway, the availability of mutant mouse lines for all lipases, and the discovery of specific lipase inhibitors ([193,194] and reviewed in [195]) represent a highly topical opportunity to aim for a quantitative description of lipolysis and its regulatory network. Developing a mathematical model of lipolysis will help to accurately dissect the relative contribution of the individual lipases to each step of acylglycerol hydrolysis.

A deeper understanding of the interactome governing lipolysis might also pave new ways for drug discovery. In recent years, dozens of small molecules or peptides targeting protein-protein interactions have reached clinical development, predominantly for the treatment of cancer [196]. Likewise, modifying specific interactions within the lipolysome pharmacologically might represent a powerful strategy to combat diseases associated with dysregulated neutral lipid catabolism.

## Abbreviations

2-AG	2-arachidonoyl glycerol
AMPK	5’ adenosine monophosphate-activated protein kinase
ABHD5	alpha/beta-hydrolase domain containing protein 5
ADP	adenosine-diphosphate
ADRP	adipose differentiation related protein
ARF-1	ADP-ribosylation factor 1
AT	adipose tissue
ATGL	adipose triglyceride lipase
ATP	adenosine-triphosphate
Bcl2	B-Cell CLL/Lymphoma 2
cAMP	cyclic adenosine monophosphate
CBRs	cannabinoid receptors
CE	cholesteryl ester
CGI-58	comparative gene identification-58
CHGB	chromogranin B
ChREBP	carbohydrate response element binding protein
CIDEC	cell death-inducing DNA fragment factor 40/45-like effector C
CL 316,243	β_3_ adrenoceptor agonist
COPI	coatomer protein I
DAG	diacylglycerol
DNL	de novo lipogenesis
ELOVL6;	elongase of long chain fatty acids family 6
ER	endoplasmic reticulum
ERK	extracellular signal-regulated kinase
FA	fatty acid
FABP	fatty acid-binding protein
FSP-27	fat-specific protein-27
G0S2	G0/G1 switch gene 2
GBF1	Golgi Brefeldin A resistance factor 1
GH	growth hormone
HIG-2	hypoxia-inducible gene-2
HILPDA	hypoxia-inducible LD-associated protein
HSL	hormone-sensitive lipase
IC_50_	half maximal inhibitory concentration
iPLA2-zeta	Ca^2+^-independent phospholipase A2-zeta
KIFC3	Kinesin Family Member C3
KO	knockout
LC3	microtubule-associated proteins 1A/1B light chain 3B
LC3-interacting region	LC3-interacting region
LD	lipid droplet
MAG	monoacylglycerol
MGL	MAG lipase
NCL	nucleolin
NLSD-M/-I	neutral lipid storage disease with myopathy/with ichthyosis
p97/VCP	partner p97 subunit/valosin containing protein
PAT	perilipin/ADRP/TIP47
PEDF	pigment epithelium-derived factor
PEX5	peroxisome biogenesis factor 5
PKA	protein kinase A
PLIN	perilipin
PNPLA	patatin-like phospholipase domain containing
PPAR	peroxisome proliferator-activated receptor
PTRF	polymerase I and transcript release factor
PTS1R	peroxisome target sequence 1 receptor protein
RNA	ribonucleic acid
RNAi	RNA interference
SND1	Staphylococcal nuclease and tudor domain containing 1
StAR	steroidogenic acute regulatory protein
TAG	triacylglycerol
TIP-47	tail-interacting protein of 47 kDa
UBXD8	ubiquitin regulatory X domain-containing protein 8
VIM	Vimentin
